# Plasma hyaluronan, hyaluronidase activity and endogenous hyaluronidase inhibition in sepsis: an experimental and clinical cohort study

**DOI:** 10.1186/s40635-021-00418-3

**Published:** 2021-10-11

**Authors:** Jaap van der Heijden, Constantinos Kolliopoulos, Paul Skorup, Marko Sallisalmi, Paraskevi Heldin, Michael Hultström, Jyrki Tenhunen

**Affiliations:** 1 Department of Surgical Sciences, Anesthesiology and Intensive Care, Uppsala University, 75185 Uppsala, Sweden; 2 Department Medical Biochemistry and Microbiology, Uppsala University, Uppsala, Sweden; 3 Department of Medical Sciences, Section of Infectious Diseases, Uppsala University, Uppsala, Sweden; 4 Division of Anaesthesia and Intensive Care Medicine, Intensive Care Units, Department of Surgery, Helsinki University Hospital, Helsinki, Finland; 5 Department of Medical Cell Biology, Integrative Physiology, Uppsala University, Uppsala, Sweden

**Keywords:** Hyaluronan, Hyaluronidase, Hyaluronidase inhibitor, Sepsis, Pancreatitis, Glycocalyx, Sheddase

## Abstract

**Background:**

Plasma hyaluronan concentrations are increased during sepsis but underlying mechanisms leading to high plasma hyaluronan concentration are poorly understood. In this study we evaluate the roles of plasma hyaluronan, effective plasma hyaluronidase (HYAL) activity and its endogenous plasma inhibition in clinical and experimental sepsis. We specifically hypothesized that plasma HYAL acts as endothelial glycocalyx shedding enzyme, sheddase.

**Methods:**

Plasma hyaluronan, effective HYAL activity and HYAL inhibition were measured in healthy volunteers (*n* = 20), in patients with septic shock (*n* = 17, day 1 and day 4), in patients with acute pancreatitis (*n* = 7, day 1 and day 4) and in anesthetized and mechanically ventilated pigs (*n* = 16). Sixteen pigs were allocated (unblinded, open label) into three groups: Sepsis-1 with infusion of live *Escherichia coli* (*E. coli*) 1 × 10^8^ CFU/h of 12 h (*n* = 5), Sepsis-2 with infusion of *E. coli* 1 × 10^8^ CFU/h of 6 h followed by 1 × 10^9^ CFU/h of the remaining 6 h (*n* = 5) or Control with no *E. coli* infusion (*n* = 6).

**Results:**

In experimental *E. coli* porcine sepsis and in time controls, plasma hyaluronan increases with concomitant decrease in effective plasma HYAL activity and increase of endogenous HYAL inhibition. Plasma hyaluronan increased in patients with septic shock but not in acute pancreatitis. Effective plasma HYAL was lower in septic shock and acute pancreatitis as compared to healthy volunteers, while plasma HYAL inhibition was only increased in septic shock.

**Conclusion:**

Elevated plasma hyaluronan levels coincided with a concomitant decrease in effective plasma HYAL activity and increase of endogenous plasma HYAL inhibition both in experimental and clinical sepsis. In acute pancreatitis, effective plasma HYAL activity was decreased which was not associated with increased plasma hyaluronan concentrations or endogenous HYAL inhibition. The results suggest that plasma HYAL does not act as sheddase in sepsis or pancreatitis.

**Supplementary Information:**

The online version contains supplementary material available at 10.1186/s40635-021-00418-3.

## Introduction

Sepsis is a life-threatening syndrome with high incidence, morbidity and mortality [1]. The pathophysiology is complex and still largely unknown making sepsis a challenge for clinicians and researchers alike. Current therapeutic options are limited and treatment is mainly based on early detection, broad-spectrum antibiotics, fluid resuscitation, vasopressors and organ support [2].

Plasma concentrations of hyaluronan are up to twenty times higher in septic patients compared with healthy individuals [3] and correlate with organ dysfunction [4] and mortality [5, 6]. The exact mechanisms leading to or any potential consequences of high plasma hyaluronan are poorly understood. Hyaluronan is a long non-sulfated polysaccharide composed of repeated disaccharide units of glucuronic acid and N-acetyl-glucosamine with a molecular weight that extends up to several million Daltons. The functional effects of hyaluronan are size dependent; high molecular weight hyaluronan (HMW-HA > 500 kDa) is anti-inflammatory, whereas fragmented hyaluronan is pro-inflammatory and pro-angiogenic [7, 8]. HMW-HA is very hygroscopic and one gram can bind up to 6 l of water [9]. An average human body contains 15 g of hyaluronan, which is unevenly distributed and mostly found in the skin (56%), skeleton and connective tissues (27%). Up to a third of the total hyaluronan content is turned-over each day, which means that small changes in either the production, transport or degradation can have major impact in tissue fluid balance and edema formation [10].

The metabolism of hyaluronan in humans is mediated through synthetic enzymes (HAS1–3), catabolic hyaluronidases (HYAL) and endogenous HYAL inhibitors [11–13]. Hyaluronan is synthesized at the plasma membrane and exported into the extracellular matrix. Hyaluronan is degraded in tissue by HYAL and reactive oxygen species or transported via the lymphatic system and degraded by HYAL in lymph nodes. Excess of hyaluronan enters the circulation and is removed mainly by the liver through HYAL. The HYAL family was first reported as “Spreading Factor” due to the ability to break down hyaluronan in the extracellular matrix and, therefore, increasing the invasiveness of bacteria and venoms [14, 15]. Little is known about endogenous HYAL inhibitors but a predominant HYAL inhibitor is hypothetically a 120 kDa member of the Inter-alpha-Inhibitor protein (IαIp) [16].

Several hypotheses have been proposed to explain increased plasma hyaluronan concentrations in sepsis. First, viral and bacterial infection and inflammatory mediators associated with sepsis (TNF-α, interleukin-1 and growth factors) increase hyaluronan production [17, 18]. Second, increased lymphatic outflow mobilizes more hyaluronan from the interstitium in experimental sepsis [19]. Decreased HYAL activity in either tissue, lymphatics and liver could lead to increased plasma hyaluronan concentration [17]. Finally, the endothelial surface layer, or glycocalyx, is damaged during sepsis. The glycocalyx is rich in hyaluronan and sepsis-induced shredding of this layer could contribute to increased plasma hyaluronan concentration [20]. Injection of HYAL has been shown to induce degradation of glycocalyx [21] and circulating HYAL is, therefore, considered as a potential endogenous sheddase [22, 23]. Any potential role of HYAL and its’ endogenous plasma inhibitors in sepsis is unknown.

The aim of this study was to investigate how plasma HYAL activity and plasma HYAL inhibition correlates with hyaluronan during systemic inflammation in a porcine *E. coli* sepsis model and in clinical human blood samples in sepsis or non-infectious inflammation in pancreatitis. We specifically hypothesized that plasma HYAL acts as an endothelial glycocalyx shedding enzyme.

## Materials and methods

### Preclinical experimental study

#### Anaesthesia and instrumentation

Sixteen healthy Swedish landrace-breed piglets (24.6 ± 1.5 kg) were used in the study. The animals had free access to water and food until transportation to the facility. All animals were given 50 mg xylazine intramuscularly before transport to minimize stress. At arrival, the animals were weighed and tiletamine–zolazepam 6 mg/kg and xylazine 2.2 mg/kg were given intramuscularly. The ear vein was cannulated and 20 mg of morphine was given. Anaesthesia was maintained using continuous intravenous infusion of sodium–pentobarbital 8 mg/kg/h, morphine 0.48 mg/kg/h and rocuronium bromide 1.5 mg/kg/h. A preload bolus of 20 ml/kg Ringer's Acetate was given followed by a continuous infusion of 2 ml/kg/h glucose 25 mg/ml. The total fluid administration, excluding interventional boluses, was 4.26 ml/kg/h. The airway was secured by tracheostomy followed by volume-controlled ventilation with tidal volumes of 6 ml/kg, inspiratory-to-expiratory ratio of 1:2, fractional inspired oxygen (FiO_2_) 0.30, positive end-expiratory pressure (PEEP) 5 cmH_2_O, and respiratory rate 25/min.

A urine catheter was placed through a vesicostomy to monitor hourly urine production. The right external jugular vein was catheterized with a pulmonary arterial catheter and central venous catheter to monitor cardiac index (CI), mean pulmonary arterial pressure (MPAP) and pulmonary capillary wedge pressure (PCWP). The right cervical artery was catheterized in to measure blood pressure and to collect blood samples. A 45–60 min of stabilization was allowed to achieve normoventilation with an arterial partial pressure of carbon dioxide (PaCO_2_) between 35 and 45 mmHg (5.0 and 5.5 kPa) by adjusting the respiratory rate. Body temperature was maintained between 38.0 and 39.5 °C using a warming mattress and blankets.

#### Induction of sepsis

Sepsis was induced by infusion of live *Escherichia coli* (*E. coli*, strain B09–11822) as previously reported [24]. We chose to use *E. coli,* because it does not express HYAL [25]. The bacterial infusion was replaced every second hour to keep the bacteria in a logarithmic growth phase. Sixteen animals were allocated into three groups (unblinded, open label): Sepsis-1 (*n* = 5, infusion of *E. coli* 1 × 10^8^ CFU/h during 12 h), Sepsis-2 (*n* = 5, infusion of *E. coli* 1 × 10^8^ CFU/h during 6 h followed by infusion of 10 × 10^8^ CFU/h during the remaining 6 h) and Control (*n* = 6, infusion of equal amount of 0.9% saline). We used daily two parallel animals consecutively allocated to each group.

Respiratory and circulatory parameters were followed continuously and recorded every hour. Blood samples (EDTA), arterial blood gas and mixed venous blood gas (SvO_2_) were taken hourly. Urine samples and blood samples for bacterial count were collected every 3 h. Total blood loss was estimated to be less than 10% of the circulating blood volume (Additional file 1: Fig. S1, time-line of preclinical experimental design).

Respiratory and circulatory interventions were protocolized according to current intensive care practice (excluding antibiotics treatment) and summarized in Additional file 2: Table S1. At the experimental endpoint the animals were killed by a direct injection of potassium chloride in the central venous line while under anaesthesia.

### Clinical study

Healthy volunteers (*n* = 20), patients with septic shock (*n* = 17) and acute alcoholic pancreatitis (*n* = 7) were enrolled from September 2008 to September 2009 at the Helsinki University Central Hospital. All data for patients with acute pancreatitis is previously unpublished, while the distribution of molecular weight for hyaluronan in plasma among healthy volunteers’ and septic shock patients’ were previously published [3]. Inclusion criteria for patients with septic shock were: age ≥ 18 years, ≥ 2 of SIRS criteria fulfilled, proven or highly suspected infection consistent with septic shock and norepinephrine therapy of at least 0.1 mcg/kg/min in spite of adequate fluid resuscitation to maintain systemic arterial pressure (SAP) above 90 mmHg. Inclusion criteria for patients with acute pancreatitis were: age ≥ 18 years, ≥ 2 of SIRS criteria fulfilled, pancreatitis confirmed by a computer tomography in the absence of biliary stones and a history of excessive consumption of alcoholic beverages (ethanol). Blood samples were taken within 24 h (day 1) of admission to the ICU and 72 h (day 4) thereafter. Plasma samples were collected in K2-EDTA-tubes and centrifuged at 18 °C for 15 min (2500 g) and stored at − 80 °C for further analysis.

### Quantification of hyaluronan, effective HYAL activity and endogenous HYAL inhibition in plasma

We quantified hyaluronan using an ELISA-like assay as described earlier [26]. An assay was adapted and optimized to measure effective HYAL activity [27] and the HYAL inhibition assay was based on the HYAL activity protocol with adjustments [16, 28, 29]. In detail, please see Additional file 3: text analysis.

### Statistics

We chose to use a convenience sample size with minimal reasonable number of animals for the experimental study, since no prior data of plasma HYAL activity and plasma HYAL inhibition in septic pigs were available. Statistical analyses were preformed using IBM® SPSS® statistics version 23 (SPSS, Inc., Chicago, IL, USA). Normality of distribution of continuous variables was tested by Shapiro–Wilk test and visually assessed using histograms. When necessary, the Expectation Maximization method for missing data was applied. One-way ANOVA or Kruskal–Wallis test were used to compare the groups. Wilcoxon signed-rank test was used to compare groups over time. Two-way repeated measures ANOVA was used to compare differences within and between the groups over time. If relevant post hoc analysis was performed with the Bonferroni correction or Mann Whitney *U* tests. Spearman’s rank-order test was used for correlation analysis. For within-subjects correlation we used the Bland Altman method [30]. The statistical tests were used according to the distribution of the data. A value of *p* < 0.05 was considered statistically significant.

## Results

### Porcine sepsis: bacterial infusion, respiratory and circulatory variables

**Fig. 1:**
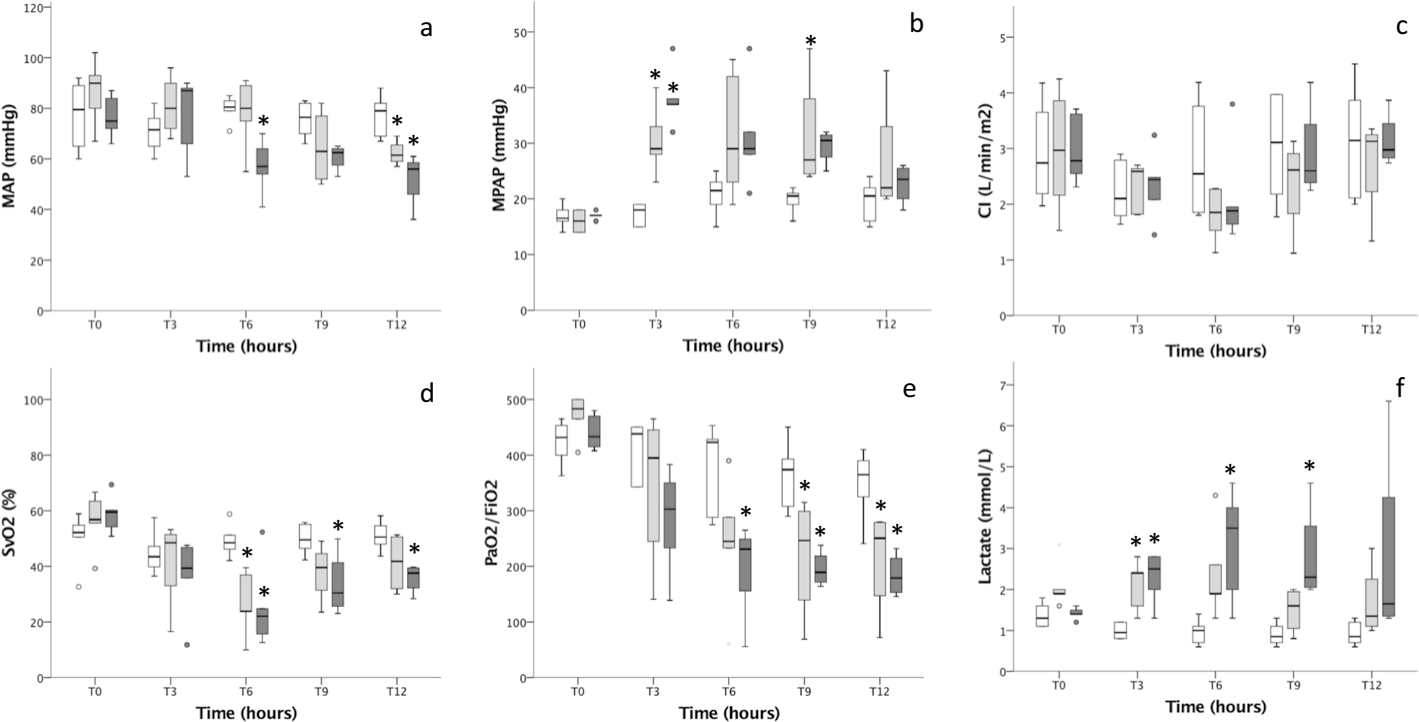
**a**–**f** Preclinical experiments. Hemodynamic and respiratory changes during the preclinical experiments from start (T0) until the experimental endpoint (T12). Control (white), Sepsis-1 (light gray) and Sepsis-2 (dark gray). *p < 0.05 compared with control group, *p*-value from one-way ANOVA with Bonferroni correction. No difference found between Sepsis-1 and Sepsis-2

The bacterial load for all Sepsis-1 (T0–12) experiments and Sepsis-2 (T0–6) was 0.9 × 10^8^ CFU/h (IQR 0.73–1.43) compared with 11.8 × 10^8^ CFU/h (IQR 10.9–14.0) for Sepsis-2 (T6–12). No live bacteria were detectable in blood cultures in the control group (Additional file 1: Fig. S2). The groups were comparable at the baseline except for blood lactate being higher in Sepsis-1 group (Additional file 2: Table S2). Animals in the control group were stable in circulatory and respiratory parameters throughout the experiment. In both sepsis groups, one animal died between 6 and 7 h after infusion with live *E. coli* following circulatory and respiratory collapse despite the predetermined interventions. The 12-h sepsis experiments were characterized by decreased mean arterial pressure (MAP), SvO_2_ and PaO_2_/FiO_2_ ratio, increased arterial lactate and a transient increase in mean pulmonary arterial pressure (MPAP) as compared to baseline and controls (Fig. 1a–f). No differences were found between Sepsis-1 and Sepsis-2. The requirement for fluid resuscitation was comparable in Sepsis-1 and Sepsis-2 (2.5 ml/kg/h (IQR 0.6–7.6) and 3.8 ml/kg/h (IQR 2.5–15.3, *p* = 0.175). Vasopressor was needed to maintain MAP ≥ 60 in both Sepsis-1 (*n* = 3/5) and Sepsis-2 (*n* = 3/5) but no difference in vasopressor load was found between the groups (*p* = 0.746).

### Porcine sepsis: hyaluronan, effective HYAL activity and HYAL inhibition in plasma

**Fig. 2:**
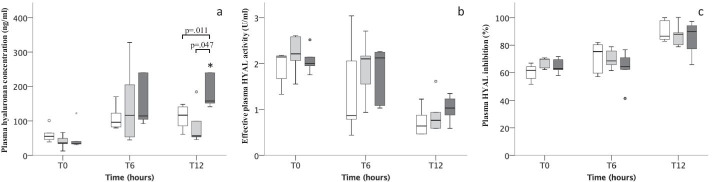
**a**–**c** Preclinical experiments. **a** Plasma hyaluronan, **b** effective plasma HYAL activity and **c** plasma HYAL inhibition for timepoint T0–6–12. **a** Plasma hyaluronan concentration increased over time in all groups (Control *p* = 0.046, Sepsis-1 and Sepsis-2 *p* = 0.043). **b** Effective plasma HYAL activity decreased during the experiment in all groups (Control *p* = 0.028, Sepsis-1 and Sepsis-2 *p* = 0.043) whereas **c** plasma HYAL inhibition increased significantly in Control and Sepsis-1 over time (Control *p* = 0.028, Sepsis-1 *p* = 0.043). Control (white), Sepsis-1 (light gray) and Sepsis-2 (dark gray). *p*-value from Mann–Whitney *U* test and Wilcoxon signed-rank test

Plasma hyaluronan concentration increased over time in all three groups and was higher in the Sepsis-2 group [158 ng/ml (IQR 147–509)] as compared to Control (117 ng/ml (IQR 80–143), *p* = 0.011) and Sepsis-1 (56 ng/ml (IQR 50–142), p = 0.047) groups after 12 h (Fig. 2a). Effective plasma HYAL activity decreased over the length of the experiment comparably in all three groups and no difference was found at 12 h (Fig. 2b). Concomitantly, activity of endogenous plasma HYAL inhibition increased over time in Control and Sepsis-1 groups (Fig. 2c). The within-subject analyses for repeated measurements showed a negative correlation between plasma hyaluronan and effective HYAL activity (rs = − 0.38, *p* = 0.026) and between effective HYAL activity and HYAL inhibition (rs = − 0.824, *p* < 0.001; Additional file 1: Fig. S3a–c). Pooled data showed a negative correlation between effective plasma effective HYAL activity and HYAL inhibition (rs = − 0.697, *p* < 0.01; Additional file 1: Fig. S4a–c) but not between plasma hyaluronan concentration and effective HYAL activity (*p* = 0.091) or plasma hyaluronan concentration and HYAL inhibition (*p* = 0.501).

### Septic shock and acute pancreatitis: hyaluronan, effective HYAL activity and HYAL inhibition in plasma

**Fig. 3:**
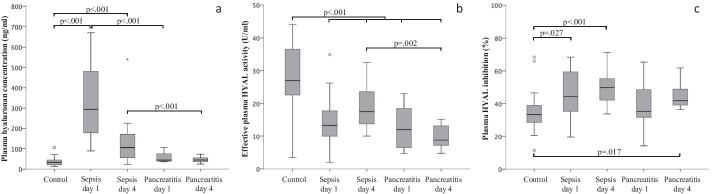
**a**–**c** Clinical experiments. **a** Plasma hyaluronan concentration, **b** effective plasma HYAL activity and **c** plasma HYAL inhibition for control, sepsis day 1 and day 4, pancreatitis day 1 and day 4. *p*-value from Mann–Whitney *U* test

Plasma hyaluronan concentrations in patients with septic shock were higher at day 1 [294 ng/ml (IQR 178–528), *p* < 0.001] and day 4 [106 ng/ml (IQR 53–179), *p* < 0.001] compared with healthy volunteers [34 ng/ml (IQR 20–46); Fig. 3]. Plasma hyaluronan concentrations in patients with acute pancreatitis at days 1 and 4 were comparable to that of healthy volunteers (*p* = 0.075 and *p* = 0.127, respectively). Plasma hyaluronan concentration was higher for septic shock compared with pancreatitis on both day 1 (*p* < 0.001) and day 4 (*p* < 0.001). Effective plasma HYAL activity in healthy volunteers [26.9 U/ml (IQR 21.6–36.7)] was higher compared to patients with septic shock at day 1 [13.2 U/ml (IQR 9.2–17.7), *p* < 0.001] and day 4 [17.5 U/ml (13.6–23.6), p = 0.001] and to patients with acute pancreatitis at day 1 [12.0 U/ml (IQR 6.4–19.6), *p* = 0.001] and day 4 [8.8 U/ml (IQR 6.7–13.6), *p* < 0.001]. Effective plasma HYAL activity was similar for patients with septic shock and pancreatitis at day 1 (*p* = 0.54) but not on day 4 (*p* = 0.002). The endogenous plasma HYAL inhibition in healthy volunteers [33.3% (IQR 28.3–39.0)] was lower compared to patients with septic shock at day 1 [44.2% (IQR 33.9–60.0), *p* = 0.027] and day 4 [49.9% (IQR 41.2–55.8), *p* < 0.001] and to patients with pancreatitis day at day 4 [41.9% (IQR 38.3–50.2), *p* = 0.017] but not day 1 [35.3% IQR 31.1–51.9), *p* = 0.542]. No within-subject correlation between plasma hyaluronan, HYAL and/or HYAL inhibition was found. Pooling data of septic shock patients and healthy volunteers showed a negative correlation between plasma hyaluronan and effective HYAL activity (rs = − 0.455, *p* = 0.001). No correlations were found for acute pancreatitis.

## Discussion

The main finding of the present study is that during experimental 12-h *E. coli* sepsis, plasma hyaluronan increased with concomitant decrease in effective plasma HYAL activity and increasing endogenous HYAL inhibition. Contrary to our hypothesis, changes of plasma hyaluronan concentration, effective HYAL activity and endogenous HYAL inhibition over time were not specifically associated to *E. coli* sepsis. Rather, general anaesthesia, surgical instrumentation and fluid infusions alone induced comparable effects in the control group. Second, in clinical septic shock of variable microbial origin, high plasma hyaluronan concentration was also associated with lower HYAL activity and higher endogenous HYAL inhibition as compared to healthy volunteers. However, in acute pancreatitis plasma hyaluronan concentration remained low, while effective HYAL activity decreased.

Baseline levels of plasma hyaluronan in the experimental study are consistent with earlier publications [3–6]. Six and twelve hours after starting bacterial infusion the plasma hyaluronan concentration were higher in all groups compared with baseline values. Sterile injury such as surgical instrumentation alone can alter hyaluronan production and metabolism [31]. Furthermore, a rapid infusion of crystalloids similar to the preload bolus in this study showed increased plasma hyaluronan in humans [32]. Finally, preparation was done under clean but non-sterile conditions. Although no positive blood cultures we found in the control group, an alternative source for infection cannot be excluded.

Decreased HYAL activity could increase plasma hyaluronan concentration through different mechanisms. In plasma, circulating HYAL is transported to liver endothelial cells by endocytosis [34, 35]. Lower HYAL activity resulting in decreased plasma hyaluronan degradation in the liver could contribute to increased plasma hyaluronan. One may also speculate that lower HYAL activity result in accumulation of hyaluronan in the extracellular matrix. Combined with increased hyaluronan production in inflammation [17, 18] this could lead to edema formation due to the strong water binding capacity of hyaluronan. Increased lymph flow during sepsis transports the excess of hyaluronan to the lymphatic system [19], where, in normal conditions, most of the hyaluronan is degraded in lymph nodes by HYAL. Decreased HYAL activity in the lymph nodes could result in an increased drainage of hyaluronan into the circulation resulting in increased plasma concentration.

Effective plasma HYAL activity is decreased in both patients with septic shock and acute pancreatitis, while plasma hyaluronan concentration is only increased in septic shock. This suggests different mechanisms responsible for plasma hyaluronan concentrations in infectious compared to non-infectious inflammation. In addition, increased plasma hyaluronan concentration in septic shock is not caused by decreased HYAL activity alone. Rather, an imbalance between increased production, decreased degradation and altered lymph flow are likely to explain these results.

Increased levels of plasma hyaluronan in sepsis are hypothesized to originate from shedding of the glycocalyx [36] and HYAL has been proposed as a potential important sheddase responsible for damaging the glycocalyx in various clinical conditions [23]. Low effective plasma HYAL activity reported herein makes it unlikely that plasma HYAL contributes to disruption of the glycocalyx in either acute pancreatitis or septic shock. Shedding of the glycocalyx resulting in increased plasma hyaluronan concentrations is observed with several other enzymes [23] and TNF-α [37]. Importantly, our observation is limited to plasma HYAL activity and thereby we cannot exclude any role of plasma membrane associated HYAL. Interestingly, endothelial dysfunction is well described in pancreatitis [38] and if shedding of the glycocalyx is thought to be a source for circulating hyaluronan, an increase of plasma hyaluronan should be expected. However, in our study plasma hyaluronan did not increase in acute pancreatitis. This brings in question if shedding of the glycocalyx significantly contributes to increased plasma hyaluronan.

Septic shock and pancreatitis result in decreased activity of effective HYAL. Surgical instrumentation, fluid infusion and general anaesthesia combined gave similar results. Recent publications on HYAL suggest protective properties in acute inflammation. Exogenous HYAL diminishes the rolling, adhesion and recruitment of neutrophils, decreases cytokine production and limits the albumin diffusion in lung and mesenteric interstitium [39–42]. Furthermore, administration of HYAL has been shown to reduce edema in both pre- and clinical studies [43–45] and is even suggested as a treatment option for COVID-19 [46]. Future research on HYAL and septic shock, acute pancreatitis and major surgery are, therefore, of high interest.

### Limitations

Our study has several limitations. The duration of our 12-h sepsis model might be not long enough to induce plasma hyaluronan concentrations as high as seen in clinical sepsis. Hyaluronan, HYAL activity and HYAL inhibition in plasma were analyzed at two timepoint during ongoing sepsis (T6 and T12). Its is therefor unclear if HYAL activity increases in plasma during early sepsis before HYAL inhibition increases. The small group size in both the preclinical and clinical experiments limit the interpretation of the results. Finally, plasma hyaluronan, HYAL activity and its endogenous inhibition might not accurately reflect any potential changes in the extracellular space or lymphatic system, limiting the generalization of our findings.

## Conclusion

Sepsis but also anaesthesia, surgical procedures and fluid infusions are associated with increasing endogenous plasma HYAL inhibition and decreasing effective plasma HYAL activity, whereas acute pancreatitis is only associated with decreased effective plasma HYAL activity. Endogenous plasma HYAL inhibition may regulate plasma HYAL activity in select infections (*E. coli*) and in inflammation (post-surgery, anaesthesia, fluid infusions). Changes in effective plasma HYAL activity alone do not explain plasma hyaluronan concentrations in bacteremia/sepsis but may, nevertheless, contribute. Increased plasma hyaluronan concentrations in sepsis are most likely caused by increased production or altered lymph flow. Plasma HYAL most likely cannot be considered as an important sheddase.

## Supplementary Information

**Additional file 1: Figure S1.** experiments. Timeline of preclinical exPreclinical perimental design. ABG: arterial blood gas, VBG: mixed venous blood gas. **Figure S2a-b.** Preclinical experiments. Characteristics of the bacteremia-sepsis model. (a) bacterial infusion in CFU/hour and (b) blood cultures showing CFU per ml blood during bacterial infusion. Control (white; no bacteria infused, no bacteria found in blood cultures), Sepsis-1 (light gray) and Sepsis-2 (dark gray). **Figure S3 a-c.** Preclinical experiments. (a) Negative within-subject correlation between plasma hyaluronan concentration and effective plasma HYAL activity (r= -.38, p=.026) and (b) effective plasma HYAL activity and HYAL inhibition (r= -.824, p<.001) but not for (c) plasma hyaluronan concentration and HYAL inhibition (p=.506). Each line represents a linear regression between three values (T=0, 6 and 12) of individual experiments from which a pooled within-subject correlation is calculated according to the Bland Altman method. The experimental groups  (control, Sepsis-1 and Sepsis-2) are shown separetely to facilitate the visualization of each individual regression line. **Figure S4 a-c.** Preclinical experiments. Pooled data of septic shock patients and healthy volunteers showing correlation between (b) effective plasma hyaluronidase activity and hyaluronidase inhibition. No correlation found between (a) plasma hyaluronan concentration and  effective hyaluronidase activity and  (c) plasma hyaluronan concentration and  hyaluronidase inhibition. Correlation and p-value from Spearman’s rank-order test.

**Additional file 2: Table S1.** Preclinical experiments. Experimental interventions for circulatory and respiratory variables, glucose and temperature. **Table S2.** Preclinical experiments. Baseline conditions, no significant differences between the groups unless for higher lactate in Sepsis-1 compared with Control (p=.020) and Sepsis-2 (p=.039). *p-value from one-wau ANOVA with Bonferroni correction, †p-value from one-way ANOVA, ‡p-value from Kruskal-Wallis test

**Additional file 3:** Analysis.

## Data Availability

The datasets used and/or analyzed during the current study are available from the corresponding author on reasonable request.

## Abbreviations

ANOVA: Analysis of variance
APACHE II: Acute Physiology and Chronic Health Evaluation
CFU: Colony-forming unit
CI: Cardiac index
*E. coli*: *Escherichia coli*
EDTA: Ethylenediaminetetraacetic acid
ELISA: Enzyme-linked immunoassay
FiO_2_: Fractional inspired oxygen
HA: Hyaluronan
HAS: Hyaluronan synthases
HMW-HA: High molecular weight hyaluronan
HYAL: Hyaluronidase
IαIp: Inter-alpha-inhibitor protein
MAP: Mean arterial pressure
MPAP: Mean pulmonary arterial pressure
PaCO_2_: Arterial partial pressure of carbon dioxide
PaO_2_: Arterial partial pressure of oxygen
PaO_2_/FiO_2_: Arterial partial pressure of oxygen/fractional inspired oxygen
PCWP: Pulmonary capillary wedge pressure
PEEP: Positive end-expiratory pressure
PVRI: Pulmonary vascular resistance index
SaO_2_: Arterial oxygen saturation
SIRS: Systemic inflammatory response syndrome
SOFA score: Sequential organ failure assessment score
SvO_2_: Mixed venous oxygen saturation
SVRI: Systemic vascular resistance index
TNF-α: Tumor necrosis factor alpha
