# Supplementary material for: Plasma hyaluronan, hyaluronidase activity and endogenous hyaluronidase inhibition in sepsis: an experimental and clinical cohort study
Source: Intensive Care Med Exp. 2021 Oct 11;9:53. doi: 10.1186/s40635-021-00418-3 (PMC8502523; doi:10.1186/s40635-021-00418-3)
Supplement: Supplementary file 1 — Additional file 1: Figure S1. experiments. Timeline of preclinical exPreclinical perimental design. ABG: arterial blood gas, VBG: mixed venous blood gas. Figure S2a-b. Preclinical experiments. Characteristics of the bacteremia-sepsis model. (a) bacterial infusion in CFU/hour and (b) blood cultures showing CFU per ml blood during bacterial infusion. Control (white; no bacteria infused, no bacteria found in blood cultures), Sepsis-1 (light gray) and Sepsis-2 (dark gray). Figure S3 a-c. Preclinical experiments. (a) Negative within-subject correlation between plasma hyaluronan concentration and effective plasma HYAL activity (r= -.38, p=.026) and (b) effective plasma HYAL activity and HYAL inhibition (r= -.824, p<.001) but not for (c) plasma hyaluronan concentration and HYAL inhibition (p=.506). Each line represents a linear regression between three values (T=0, 6 and 12) of individual experiments from which a pooled within-subject correlation is calculated according to the Bland Altman method. The experimental groups (control, Sepsis-1 and Sepsis-2) are shown separetely to facilitate the visualization of each individual regression line. Figure S4 a-c. Preclinical experiments. Pooled data of septic shock patients and healthy volunteers showing correlation between (b) effective plasma hyaluronidase activity and hyaluronidase inhibition. No correlation found between (a) plasma hyaluronan concentration and effective hyaluronidase activity and (c) plasma hyaluronan concentration and hyaluronidase inhibition. Correlation and p-value from Spearman’s rank-order test. [file 40635_2021_418_MOESM1_ESM.pptx]

## Slide 1
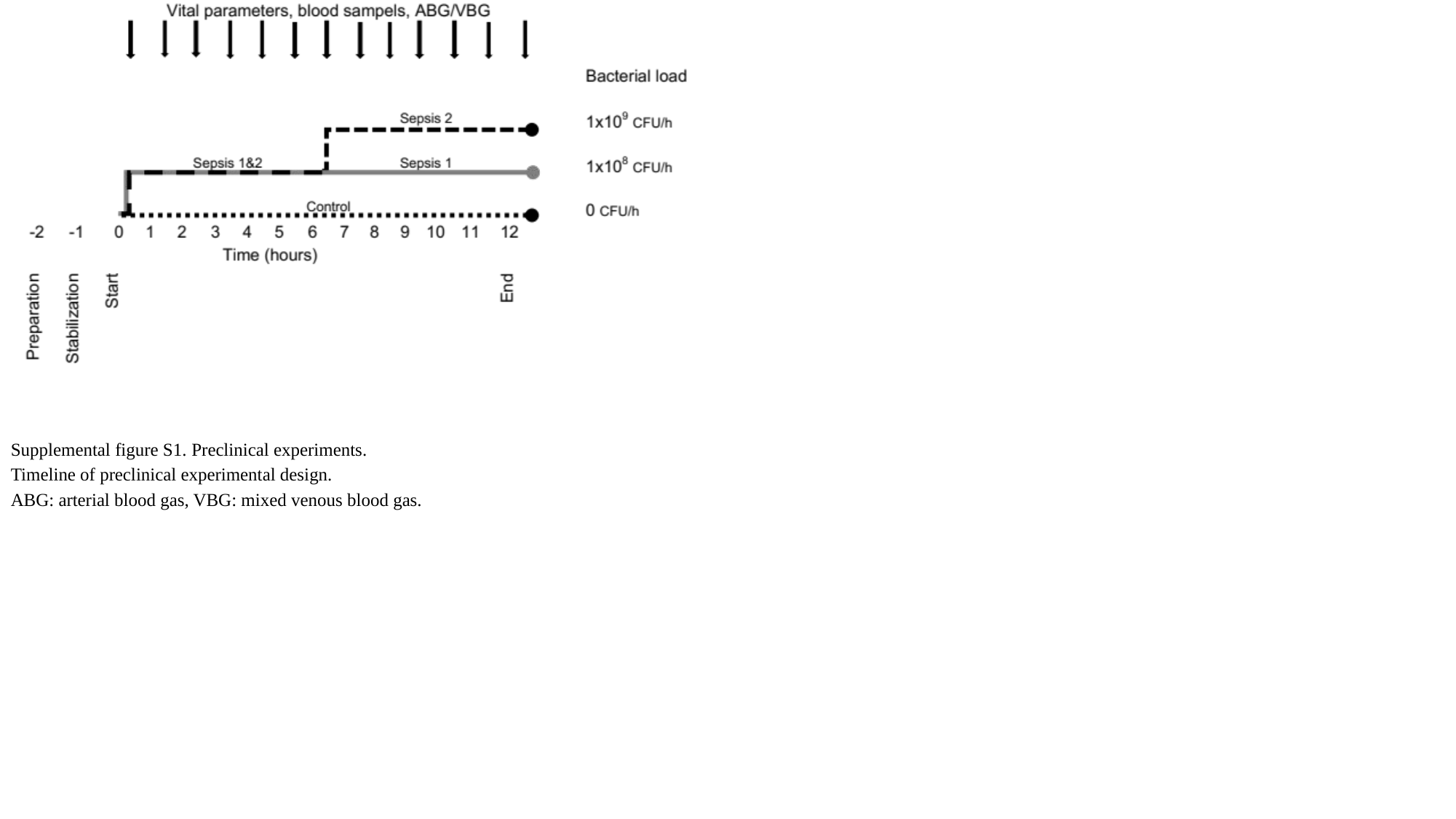

Supplemental figure S1. Preclinical experiments.
Timeline of preclinical experimental design.
ABG: arterial blood gas, VBG: mixed venous blood gas.

## Slide 2
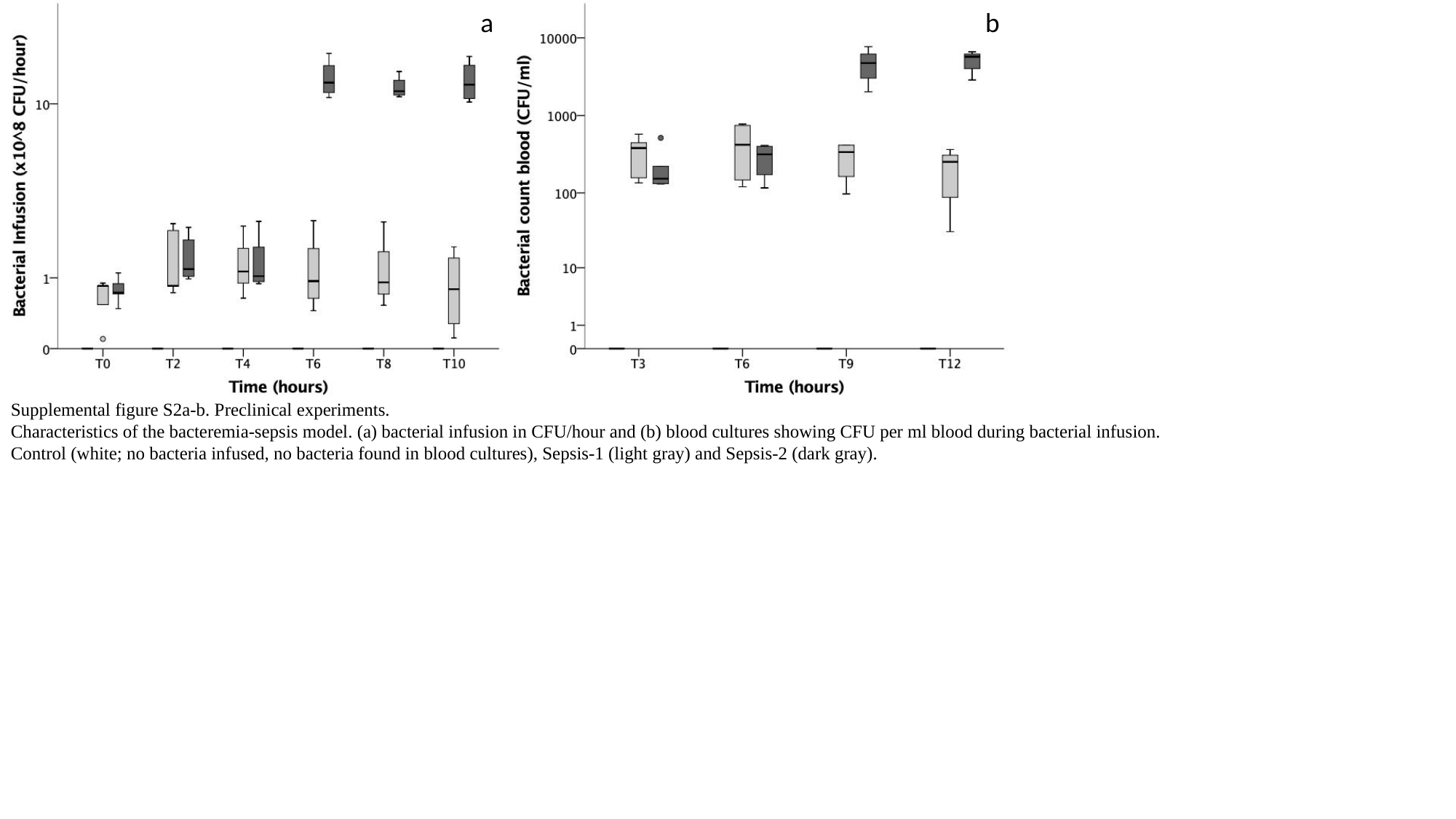

a
b
Supplemental figure S2a-b. Preclinical experiments.
Characteristics of the bacteremia-sepsis model. (a) bacterial infusion in CFU/hour and (b) blood cultures showing CFU per ml blood during bacterial infusion.
Control (white; no bacteria infused, no bacteria found in blood cultures), Sepsis-1 (light gray) and Sepsis-2 (dark gray).

## Slide 3
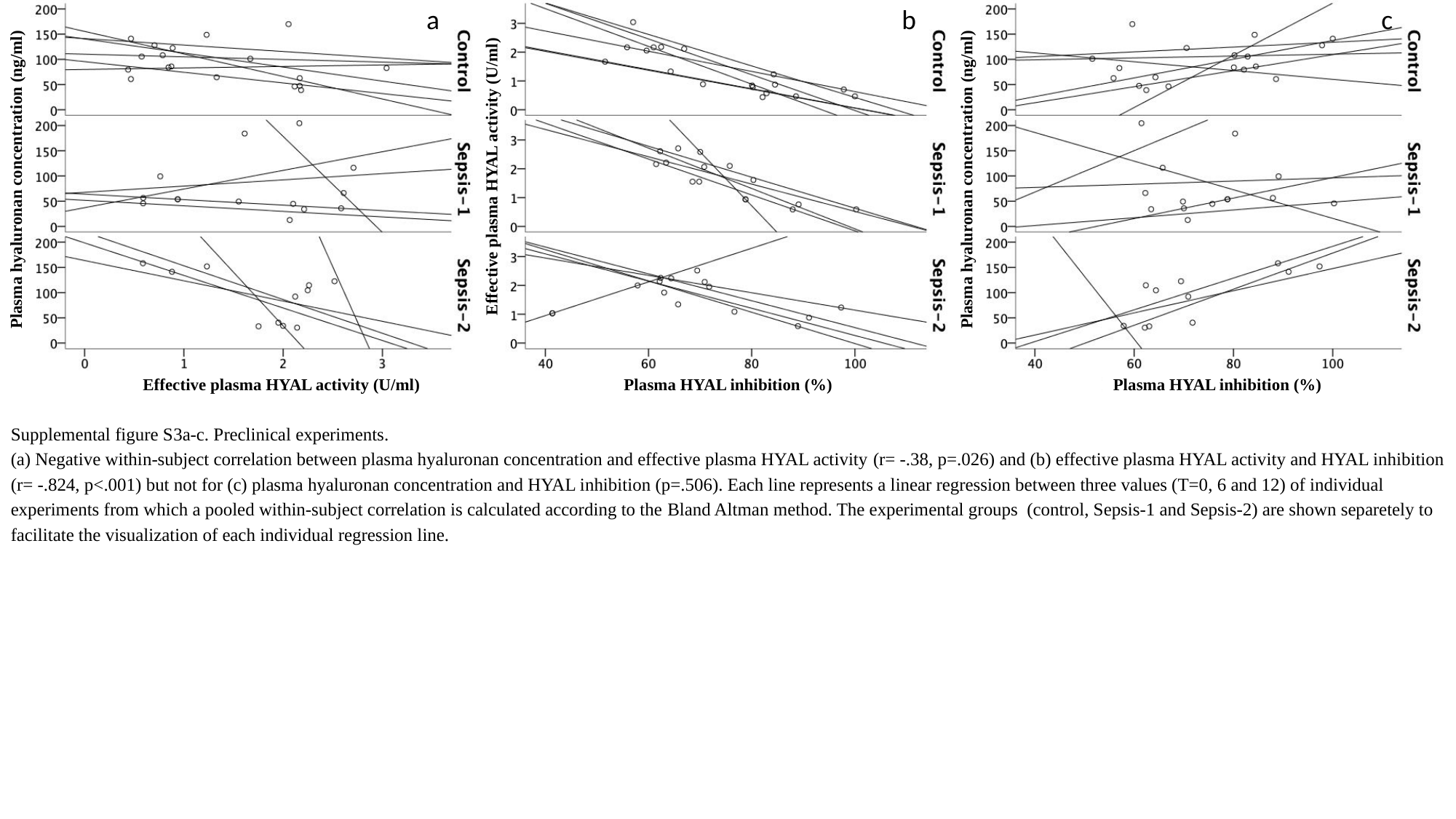

a
b
c
Effective plasma HYAL activity (U/ml)
Plasma hyaluronan concentration (ng/ml)
Plasma hyaluronan concentration (ng/ml)
Effective plasma HYAL activity (U/ml)
Plasma HYAL inhibition (%)
Plasma HYAL inhibition (%)
Supplemental figure S3a-c. Preclinical experiments.
(a) Negative within-subject correlation between plasma hyaluronan concentration and effective plasma HYAL activity (r= -.38, p=.026) and (b) effective plasma HYAL activity and HYAL inhibition (r= -.824, p<.001) but not for (c) plasma hyaluronan concentration and HYAL inhibition (p=.506). Each line represents a linear regression between three values (T=0, 6 and 12) of individual experiments from which a pooled within-subject correlation is calculated according to the Bland Altman method. The experimental groups (control, Sepsis-1 and Sepsis-2) are shown separetely to facilitate the visualization of each individual regression line.

## Slide 4
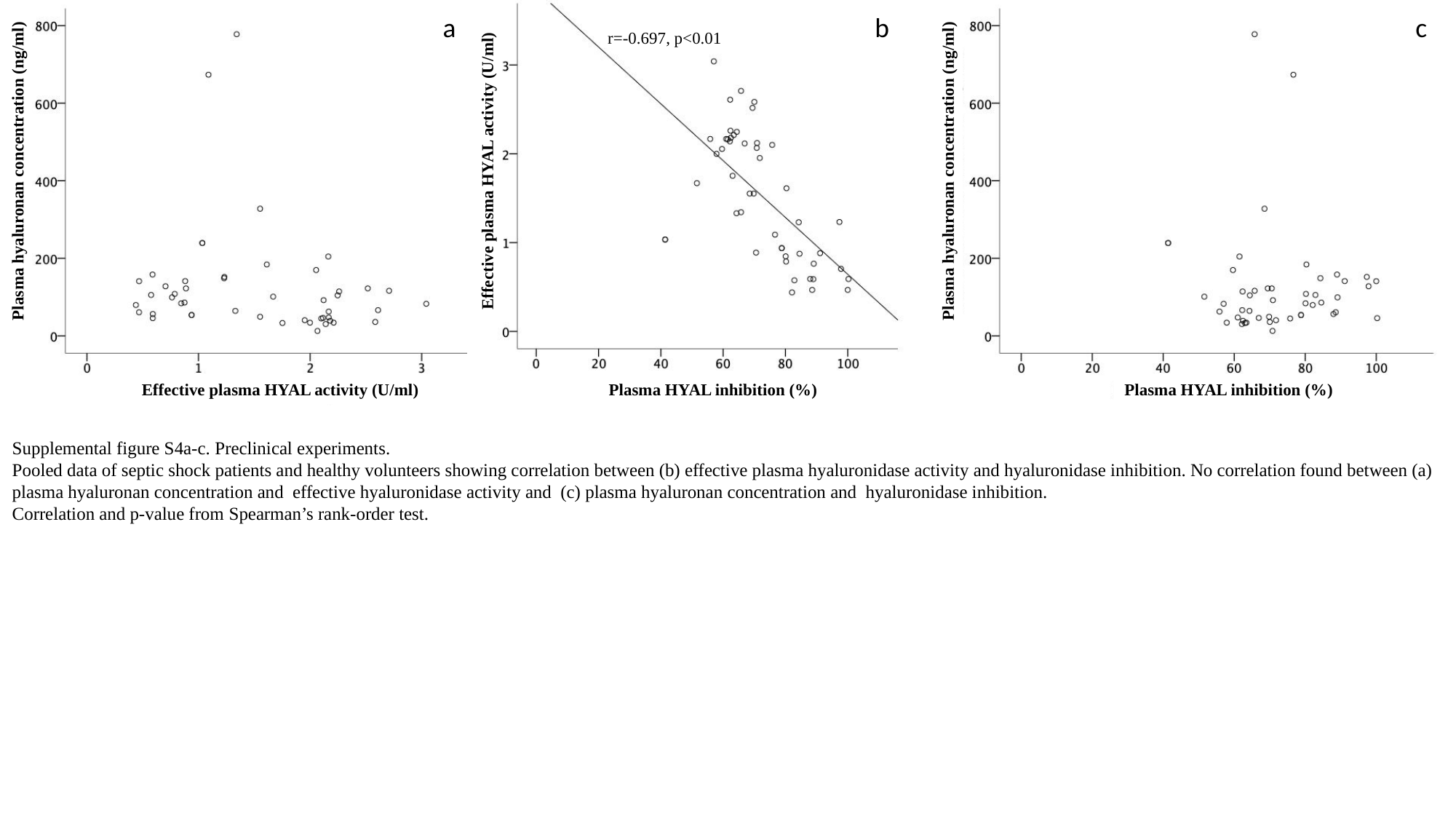

a
b
c
r=-0.697, p<0.01
Plasma hyaluronan concentration (ng/ml)
Effective plasma HYAL activity (U/ml)
Plasma hyaluronan concentration (ng/ml)
Effective plasma HYAL activity (U/ml)
Plasma HYAL inhibition (%)
Plasma HYAL inhibition (%)
Supplemental figure S4a-c. Preclinical experiments.
Pooled data of septic shock patients and healthy volunteers showing correlation between (b) effective plasma hyaluronidase activity and hyaluronidase inhibition. No correlation found between (a) plasma hyaluronan concentration and effective hyaluronidase activity and (c) plasma hyaluronan concentration and hyaluronidase inhibition.
Correlation and p-value from Spearman’s rank-order test.
