# Supplementary material for: Plasma hyaluronan, hyaluronidase activity and endogenous hyaluronidase inhibition in sepsis: an experimental and clinical cohort study
Source: Intensive Care Med Exp. 2021 Oct 11;9:53. doi: 10.1186/s40635-021-00418-3 (PMC8502523; doi:10.1186/s40635-021-00418-3)
Supplement: Supplementary file 2 — Additional file 2: Table S1. Preclinical experiments. Experimental interventions for circulatory and respiratory variables, glucose and temperature. Table S2. Preclinical experiments. Baseline conditions, no significant differences between the groups unless for higher lactate in Sepsis-1 compared with Control (p=.020) and Sepsis-2 (p=.039). *p-value from one-wau ANOVA with Bonferroni correction, †p-value from one-way ANOVA, ‡p-value from Kruskal-Wallis test [file 40635_2021_418_MOESM2_ESM.docx]

| **Parameter** | **Threshold** | **Intervention** |
| --- | --- | --- |
|  |  |  |
| **Circulatory** |  |  |
| MAP | < 60 mmHG | 1. Fluid bolus Ringer Acetate 15 ml/kg  ongoing bolus or >2^nd^ bolus/h: step 2. |
|  |  | 2. Norepinephrine 20 mcg/ml  start or increase norepinephrine infusion  0.07 → 0.13 → 0.20 → 0.26 → 0.33 → 0.40 mcg/kg/min |
| MAP | > 80 mmHg | Ongoing norepinephrine, decrease infusion  0.40 → 0.33 → 0.26 → 0.20 → 0.13 → 0.07 mcg/kg/min |
| CO | CO < 1.50 L/min | Fluid resuscitation with bolus Ringer Acetate 15 ml/kg |
| CO and diuresis | CO < 1.75 L/min and  Diuresis < 0.5 ml/kg/h | Fluid resuscitation with bolus Ringer Acetate 15 ml/kg |
|  |  |  |
| **Respiratory** |  |  |
| PaO_2_ | < 8 kPa | Increase FiO_2_ 0.3 → 0.5 → 0.7 → 0.9 → 1.0 |
| PaCO_2_ | > 6.5 kPa | Increase respiratory rate with 3/min |
|  |  |  |
| **Other** |  |  |
| Glucose | < 4 mmol/L | Bolus of 10ml with glucose 30% |
| Temperature | < 38.0 °C | Add 3 sheets, start heater |
|  | > 39.5 °C | Remove 3 sheets, turn off heater |

Supplemental table S1. Preclinical experiments.

Experimental interventions for circulatory and respiratory variables, glucose and temperature.

|  | **Control (n=6)** | **Sepsis-1 (n=5)** | **Sepsis-2 (n=5)** | **p-value**^†^ |
| --- | --- | --- | --- | --- |
|  | Mean (SD) | Mean (SD) | Mean (SD) |  |
| Weight (kg) | 24.1 (0.4) | 24.7 (0.6) | 25.3 (0.9) | .405 |
| SaO_2_ (%) | 96.4 (0.4) | 96.7 (0.7) | 97 (0.4) | .708 |
| PaO_2_/FiO_2_ | 424 (38) | 471 (39) | 441 (33) | .147 |
| Heart rate (bpm) | 100 (33) | 106 (44) | 108 (21) | .921 |
| MAP (mmHg) | 78 (13) | 86 (13) | 77 (9) | .381 |
| SVRI (dyn s/cm5/m2) | 2178 (946) | 2650 (1509) | 1983 (460) | .599 |
| MPAP (mmHg) | 17 (2) | 16 (2) | 17 (1) | .625 |
| PVRI (dyn s/cm5/m2) | 310 (73) | 274 (54) | 311 (71) | .606 |
| CI (L/min/m2) | 2.9 (0.9) | 3.0 (1.1) | 3.0 (0.6) | .989 |
| SvO_2_ (%) | 50 (9) | 56 (11) | 59 (7) | .295 |
| Hemoglobin (g/l) | 99 (5) | 99 (9) | 96 (6) | .818 |
| pH | 7.48 (0.04) | 7.49 (0.05) | 7.49 (0.04) | .772 |
| Lactate (mmol/l) | 1.4 (0.3) | 2.1* (0.6) | 1.4 (0.2) | .013 |
| Base excess (mmol/l) | 5.8 (2.3) | 5.4 (3.4) | 6.1 (2.30) | .920 |
|  | | | |  |
|  | **Control** | **Sepsis-1** | **Sepsis-2** | **p-value**^‡^ |
|  | Median (IQR) | Median (IQR) | Median (IQR) |  |
| Hyaluronan (ng/ml) | 55 (44-73) | 36 (23-58) | 34 (32-81) | .260 |
| Hyaluronidase activity (U/ml) | 2.14 (1.7-2.2) | 2.20 (2.0-2.6) | 2.00 (2.0-2.1) | .492 |
| Hyaluronidase inhibition (%) | 62 (56-64) | 69 (63-70) | 63 (62-69) | .153 |
|  |  |  |  |  |

Supplemental table S2. Preclinical experiments.

Baseline conditions, no significant differences between the groups unless for higher lactate in Sepsis-1 compared with Control (p=.020) and Sepsis-2 (p=.039).

* p-value from one-wau ANOVA with Bonferroni correction, ^†^ p-value from one-way ANOVA, ^‡^ p-value from Kruskal-Wallis test
