# Supplementary material for: Plasma hyaluronan, hyaluronidase activity and endogenous hyaluronidase inhibition in sepsis: an experimental and clinical cohort study
Source: Intensive Care Med Exp. 2021 Oct 11;9:53. doi: 10.1186/s40635-021-00418-3 (PMC8502523; doi:10.1186/s40635-021-00418-3)
Supplement: Supplementary file 3 — Additional file 3: Analysis. [file 40635_2021_418_MOESM3_ESM.docx]

**Supplemental text, Analysis**

**HA quantification**

96-well microtiter plates (Maxisorb, Nunc) were precoated with Hyaluronan-Binding Protein (HABP, 1 μg/ml in 50 mM Na_2_CO_3_, pH 9.5) and stored overnight at 4°C. The wells were rinsed with 0.05% Tween-20 PBS and blocked with 1% Bovine Serum Albumin (BSA) PBS to block any nonspecific binding. Hyaluronan standards (0-50 ng/ml) and plasma samples (EDTA, 10x dilution) were added into the wells in duplicates and incubated for 1 hour at room temperature. The plates were incubated sequentially with biotin-labeled HABP (1 mg/ml), streptavidin horseradish peroxidase (HRP, 1:1600 in 1% BSA PBS) and 3, 3′,5 ,5′-Tetramethylbenzidine (TMB) which resulted in a colour development of the wells that was terminated with 2M H_2_SO_4_. The absorbance was read at 450 nm (EnSpire Multimode Plate Reader, PerkinElmer)

**Quantification of HYAL activity in plasma**

96-well microtiter plates (Covalink, Nunc) were coated with Sulfo-NHS (0.184 mg/ml) and biotin-labeled HA (0.2 mg/ml). EDAC (0.123 mg/ml) was added and the plates were stored overnight at 4°C. Wells were equilibrated with the adequate pH buffer (0.1M formate, 0.1M NaCl, 1% Triton X-100, 5mM saccharolactone, pH 3.7 and pH 4.5 for lysosomal plasma HYAL and bovine testis/neutral-active enzymes respectively). Bovine testicular HYAL with known activity (1 to 1x10^-4^ TRU/well, diluted in pH 4.5 buffer) and the plasma samples (EDTA, 100x dilutions in pH 3.7 buffer) were added in individual wells in duplicates and incubated for 90 minutes at room temperature. Positive (no enzyme) and negative (no streptavidin-HRP) controls were included. The reaction was terminated with 6M Guanidine-HCl, and the wells were incubated with streptavidin-HRP (1:1600 in 1% BSA 0.1% Tween PBS) followed by TMB. This reaction was terminated with 2M H_2_SO_4_ and absorbance was measured at 450 nm.

**Quantification of endogenous HYAL inhibition in plasma**

96-well plates as used with the HYAL assay were prepared and stored overnight. Plasma samples (EDTA, 900x dilution) were added to a pH 7.5 buffer (50mM Hepes, pH 7.5 0.1M NaCl, 1% Triton X-100) containing 1 TRU/ml bovine testicular HYAL and 2.0mM Mg^2+^. As endogenous plasma HYAL1 is active at an acidic pH (pH optimum 3.7) the pH 7.5 buffer ensured that only the added bovine testis HYAL (bimodal distribution of activity with pH optima at pH 4.5 and 7.5) was active and could be compared with the inhibition activity of plasma samples. Addition of Mg^2+^ was needed to recover inhibition activity from plasma compared with serum. The standard curve (1 to 1x10^-4^ TRU/well, diluted in pH 7.5 buffer) and samples were added to individual wells in duplicates and incubated for 60 minutes at room temperature. The following steps were similar as the HYAL protocol. The HYAL plasma inhibition was calculated in percent as follow: inhibition (%) = 1- ((Amax-Asample)/(Amax-Amin)) where Amax, Amin and Asample where the absorbance of the wells of the positive control, the wells exposed to only bovine hyaluronidase (no inhibitor) and the samples containing both HYAL and plasma inhibitor respectively.
